# Effects of distraction on memory and cognition: a commentary

**DOI:** 10.3389/fpsyg.2014.00841

**Published:** 2014-07-29

**Authors:** Fergus I. M. Craik

**Affiliations:** Rotman Research Institute of Baycrest, Baycrest CentreToronto, ON, Canada

**Keywords:** attention, distraction, domain-general suppression, domain-specific interference, aging

## Abstract

This commentary is a review of the findings and ideas reported in the preceding nine articles on the effects of distraction on aspects of cognitive performance. The articles themselves deal with the disruptive effects of distraction on recall of words, objects and events, also on visual processing, category formation and other cognitive tasks. The commentary assesses the part played by “domain-general” suppression of distracting information and the “domain-specific” competition arising when tasks and distraction involve very similar material. Some forms of distraction are meaningfully relevant to the ongoing task, and Treisman’s (1964) model of selective attention is invoked to provide an account of findings in this area. Finally, individual differences to vulnerability to distraction are discussed; older adults are particularly affected by distracting stimuli although the failure to repress distraction can sometimes prove beneficial to later cognitive performance.

## INTRODUCTION

In our noisy world distractions are almost constantly present, competing with our attention as we attempt to focus on learning, recalling past events, or solving difficult problems. What are the factors that contribute to success or failure in blocking out such distracting information? This is the principal question asked by the researchers who contributed to the preceding articles. In this commentary I will summarize some of the main findings, highlight the common principles that unite them, and try to resolve discrepancies where they exist. The commentary will be frankly biased towards my personal view of cognition in terms of active processing operations, and will draw on findings and ideas from the older literature where they appear to make sense of current observations.

## DISTRACTION: GENERAL OR SPECIFIC?

One question that runs through a number of the articles is whether distraction impairs cognitive performance by depleting some general resource or by competing for specific representational space. That is, do we use up general attentional resources when we attempt to block out unwanted stimulation, thereby leaving less of a limited supply to fuel the main task, or is distraction specifically disruptive only when the irrelevant stimulation is qualitatively similar to task-relevant information? There is evidence in the foregoing articles for both positions. Mastroberardino and Vredeveldt (2014) had Italian children aged 8–11 years watch a 5 min video clip containing both visual and auditory details; the children were later asked questions about these details under various conditions. The children either watched a blank screen while doing the retrieval task, retrieved with eyes-closed (EC), watched a visual display of Hebrew words presented at a 1 s rate or heard Hebrew words spoken at a 1 s rate. The results were that the blank screen and EC conditions were associated with better recall of visual details than the visual and auditory distraction conditions which did not differ. Surprisingly, recall of auditory details was unaffected by the different retrieval conditions. The results for visual details show that both visual and auditory distraction impaired retrieval, suggesting that attentional resources were taken up in blocking the distracting stimuli, thereby reducing the effectiveness of retrieval. However, this “general” account does not fit the observed absence of an effect on recalling auditory details, and also fails to replicate the findings from an earlier study of adult participants (Vredeveldt et al., 2011) which showed modality-specific interference effects with very similar materials. The authors suggest that the present results might reflect the particular difficulty that children may have in focusing sustained attention on a retrieval task over time. This seems very reasonable although it is then curious why distraction had no effect on the recall of auditory details. The authors speculate that auditory details in the video used were tied in to accompanying social interactions and that this may have buffered retrieval against distraction.

Thus the article by Mastroberardino and Vredeveldt (2014) concludes that the recall of visual details benefits from the removal of either visual or auditory distraction. The generality of this conclusion is questioned, however, by the results of studies reported by Kyriakidou et al. (2014). These authors presented Cypriot children aged 6–12 years with a complex visual/auditory event lasting 10 min; the children were then interviewed about what they had experienced, either soon after the event or a week later. Half of the children were tested under EC conditions, and the other half were questioned about the event with their eyes open. The results showed that correct visual details were better recalled by the EC group (6.2 vs. 5.7 items). There was no significant effect of the EC manipulation on recall of correct auditory details, suggesting a modality-specific effect of distraction, but it is worth noting that the benefit to the EC group (4.9 vs. 4.3 items) was actually slightly greater than the difference between groups for visual details. It therefore seems preferable to conclude that the beneficial effect of EC during questioning was a general effect in this study although with greater benefits to the recall of visual details, as with Mastroberardino and Vredeveldt (2014).

The picture is complicated by the fact that Kyriakidou et al. (2014) found no effects of the EC manipulation in a second experiment, and indeed comment in a footnote that they have carried out 10 similar studies and found a beneficial effect of eye closure in only one case. The authors suggest that finding beneficial effects may depend on other environmental factors such as the length of the interview and how comfortable children are with the interviewer. The importance of such social factors is underlined by Buchanan et al. (2014) who had undergraduate participants trace their way mentally though a 3-D block matrix in response to auditory instructions. While performing this task, participants either closed their eyes, maintained eye contact with an interlocutor, maintained contact with the interlocutor wearing dark glasses, watched the interlocutor whose head was averted, or watched the interlocutor whose head was completely covered. Performance on the visual task was best in the EC condition, substantially reduced in the dark glasses conditions and greatly impaired in the eye contact conditions (Buchanan et al., 2014; Figure 2). This study makes the nice point that it is not simply irrelevant visual stimulations that interferes with what is essentially a visual working memory task (see e.g., Baddeley et al., 1975; Logie et al., 1990), but that the social and affective consequences of maintaining eye contact with another person are particularly disruptive to performance. Two interesting questions to pursue in this context are first whether eye contact would be equally disruptive to performance of complex auditory-verbal working memory tasks (e.g., “alpha span,” Craik, 1986) and second whether the effects of eye contact are essentially due to an involuntary siphoning off of general processing resources or whether the interference is more specifically affective in nature. It should also be noted that the task used by Buchanan et al. (2014) involved online visual processing, and not the retrieval of episodic events as in the studies by Mastroberardino and Vredeveldt (2014) and by Kyriakidou et al. (2014).

Rae and Perfect (2014) studied the effects of visual distraction on the retrieval of visually presented word lists in an attempt to replicate the finding by Glenberg et al. (1998) that visual distraction reduced the retrieval of mid-list items from a recently presented list of words (Experiment 5). Participants in the Rae and Perfect (2014) experiments studied lists of individual words and then attempted to recall the words orally while looking at a screen displaying either static or dynamic visual noise (Figure 1). There was also an EC condition but the results were not reported due to a coding error in the program. In Experiment 1 the authors did find that the dynamic noise condition was associated with poorer recall of mid-list items than the static noise condition, but this result was not replicated in Experiments 2 and 3. Accordingly, Rae and Perfect express considerable doubt about the claim that environmental distraction competes with the internal resources required for effortful memory retrieval.

These doubts are at first reinforced by the results reported by Craik et al. (1996). These authors found that whereas performance of a secondary task during memory *encoding* had a large detrimental effect on the later retrieval of word lists, performance of the same secondary task during the *retrieval* phase had relatively little effect on memory performance (although performance of the secondary task was impaired). Considering that the Craik et al. (1996) study involved performance of a demanding secondary task concurrently with retrieval and yet found only slight effects on memory performance, it is not surprising that Rae and Perfect (2014) also found very small effects of a distracting visual display which needed no response from the participant. Such findings of negligible effects of competing stimuli or activities on retrieval processes are particularly puzzling in light of other data showing that retrieval operations are quite costly in terms of processing resources (Craik and McDowd, 1987; Craik et al., 1996). The best explanation may be that retrieval processing is somehow protected or given priority and that any increases in processing costs are largely borne by the secondary task or by other forms of concurrent processing.

These slight effects on retrieval must be reconsidered in light of results reported by Fernandes and Moscovitch (2000), however. These researchers had participants learn a list of auditorily presented words for later free recall. Participants also performed a variety of visually presented distracting tasks concurrently with either the encoding phase or the retrieval phase. The essential finding was that performance of a secondary task during *encoding* had a substantial negative effect on later recall regardless of the qualitative nature of the secondary task, whereas performance of a secondary task during *retrieval* was disruptive to recall only when the secondary task material was similar to the material being recalled. They concluded that during encoding the memory and concurrent tasks compete for general resources, but at retrieval the competition is for material-specific representational systems. This account is in line with Rae and Perfect’s (2014) results in that little interference with recall should be expected when the distracting task (dynamic visual noise) is very different from the material being recalled (single words). By the same token it seems at first that the results of Glenberg et al. (1998) Experiment 5 are anomalous, as they did report a disruptive effect of a dynamic visual display on oral recall of words. However, the decrease in recall from the static to the dynamic display was only 0.05 (0.28–0.23) and this drop of 18% is broadly comparable to the drops of 13% reported by Fernandes and Moscovitch (2000) when participants performed a digit monitoring task during word recall, and the drop of 13% reported by Craik et al. (1996) when participants performed a visual RT task during oral word recall. It should also be noted that Wais and Gazzaley (2014, Figure 2B) report a small but significant effect of auditory distraction on the recall of visual detail, and so argue for a domain-general effect of environmental distraction on episodic retrieval. An interim summary statement might therefore be that a second source of information (either distraction or a secondary task) can disrupt retrieval, with the amount of disruption depending on such factors as the specificity of the material to be retrieved, the similarity of the secondary information to the material retrieved, the complexity or meaningfulness of the secondary information, and whether the information requires a response.

The article by Wais and Gazzaley (2014) reports a series of studies examining the effects of visual and auditory distractors on retrieval of information about visually presented objects. Two types of retrieval task were examined; in the first, participants were presented with an auditory cue word (e.g., “pumpkin”) and had to decide whether the word represented a previously presented object; in the second task, participants had to recall how many exemplars of old objects had been presented in the original display (1–4). Thus the first task is a variant of cross-modal recognition memory, and the second requires detailed visual recollection. The essential results were that visual distraction reduced recognition performance whereas auditory distraction did not. Interestingly, however, both visual and auditory distraction reduced correct recall of numbers (Wais and Gazzaley, 2014; Figure 2). The authors suggest that the effect of distraction is to reduce the *fidelity* of retrieval from long-term memory (LTM), and that limited capacity control processes attempt to resolve the difference between target information and noisy interference. These resolving operations are effective when the task is relatively easy and the distracting information qualitatively different from target information (e.g., no effect of auditory distraction on recognition memory), but are overwhelmed when the task is more difficult (e.g., recall of number) so that both visual and auditory distraction are now disruptive. This result suggests that both domain-general (resource reduction) and domain-specific (interference) factors come into play in distracting tasks, with the prevalence of each depending on such factors as task difficulty and the level of specific detail required.

Other comments on the Wais and Gazzaley (2014) article include the point that there appears to be increasing interest in the concept of *fidelity* of mental representations and how fidelity may be compromised by the aging process, both during retrieval as in the present article, but also during encoding as suggested by Benjamin (2010). The authors also stress the difference between *distraction* and *interruption*. In a distraction paradigm the non-task source of stimulation is irrelevant to performance of the main task and thus should be blocked as far as possible. In an interruption paradigm the second source of information must be attended to and often responded to as well; attentional control must therefore be managed by the executive system, with attentional resources allocated to the two tasks as optimally as possible. The two paradigms are clearly different in many respects but there may also be commonalities in that disruption of the primary task will depend on such things as the amount of resource reduction caused by the secondary activity and the similarity of operations between the main task and those needed to block or perform the secondary activity. Finally, Wais and Gazzaley (2014) relate their behavioral observations of distraction to their neural underpinnings. Their data reveal that disruption of episodic retrieval of visual information is associated with the decreased efficiency of a functional network linking the left prefrontal cortex, the hippocampus and left lateral occipital cortex. This is sophisticated work helping to illuminate the complex operations involved in memory retrieval.

## INTERACTIONS WITH AGING

The article by Wais and Gazzaley (2014) also reported some interesting age-related differences in the effects of distraction. First, comparisons between younger and older adults under visual distraction conditions showed no age difference in recognition performance but that younger participants outperformed older participants in recall of number. This interaction between age and type of test may be attributable to a differentially greater age decrement in recall as opposed to recognition (Craik and McDowd, 1987) or, as the authors prefer, to a greater age-related vulnerability to retrieval of details (number) as opposed to more general characteristics (overall recognition). Wais and Gazzaley (2014) also presented evidence for a greater susceptibility of older adults to distraction in a visual categorization task (Figure 5). The finding that older adults are more vulnerable to the effects of distraction has been documented in a series of studies by Hasher and Zacks (1988) and Hasher et al. (1999). One unexpected by-product of these studies is the finding that whereas older adults are less efficient than their younger counterparts at inhibiting unwanted stimulation, the irrelevant information may be used positively at a later time if the information is relevant to a new task. This beneficial effect of distraction is documented in the article by Weeks and Hasher (2014). Their general conclusion is that distraction is a “double-edged sword” for older adults. On the one hand their performance on a designated task is typically more disrupted by distraction than is the case for young adults, but on the other hand older adults can benefit from poorly inhibited distracting information if that information is then congruent to the performance of a later task. Weeks and Hasher point to a number of real-life situations in which older adults can make good use of poorly inhibited distracting stimuli, although presumably there is a trade-off between the negative effects of distraction on the first task and the benefits to a second congruent task. It also seems that the later benefits are largely attributable to *implicit* effects, and an interesting further question relates to the pattern of results when the second task requires explicit knowledge of the poorly inhibited distracting material.

## ATTENTION AND DISTRACTION

Hyman et al. (2014) report two intriguing and convincing studies on inattentional blindness—the phenomenon in which preoccupied people avoid obstacles yet apparently have no perceptual awareness or later memory of these obstacles. As the authors show in an ingenious experiment, people talking or texting on cell phones avoided a low-hanging branch impeding their route yet failed to register the bizarre fact that three-dollar bills had been clipped to the branch (Hyman et al., 2014, Figure 1). The authors also point out that avoiding obstacles while distracted is typically not all-or-none: “For example, a driver needs to respond differently to a large truck, a car, a bicyclist, and a pedestrian” (Hyman et al., 2014, p. 6). They suggest that such findings may be understood in terms of the differential information provided by two distinct visual processing pathways, a ventral pathway concerned with object recognition and a dorsal pathway guiding behavior although not analyzing the perceptual nature of the information (Goodale and Milner, 1992). On the assumption that the dorsal pathway is somehow more fundamental, the results of Hyman et al. (2014) may be taken to show that distracted individuals process visual information by the dorsal route, thereby enabling avoidance of the obstacle, but not fully by the ventral route, resulting in functional “blindness” for the obstacle’s characteristics.

I would like to suggest an alternative account which is that perceptual analysis is not all-or-none, but is accomplished more or less fully as a function of interactions between the salience of the perceptual input on the one hand and the person’s expectations, meaningfulness of the input and amount of attention allocated to relevant processing on the other. I am appealing here to the model of selective attention proposed by Treisman (1964). This is a “levels of analysis” view in which incoming stimuli must pass through a hierarchy of analytic “tests” running progressively from analyses concerned with physical and sensory features to later analyses concerned with object identification and semantic implication. Each test level is regarded as a signal-detection decision mechanism that incoming stimuli either pass and proceed to further analytic tests, or fail and be processed no further. The level of awareness associated with a particular input depends on the number and nature of analytic levels successfully accomplished. Treisman suggested that whether or not an incoming stimulus passes each test depends both on signal strength (a d′ variable set by the incoming stimulation) and on the criterion of importance for that specific stimulus (a ß variable set by the perceiver’s past history and current expectations). Thus by this view loud or bright stimuli will typically force their way through to conscious awareness, but important or expected stimuli (such as a person’s name) will also reach conscious awareness even when attention is diverted, by virtue of the relevant test criteria being set favorably at all times.

This model of selective attention and its associated feature of varying levels of analysis and awareness would thus account for the results of Hyman et al. (2014) by claiming that early physical features such as shape, size, and direction were analyzed by the visual–perceptual system—enough to drive avoidance behavior—but no further analyses were either necessary or relevant, leading to a failure to identify the surprising features of the obstacle. This failure to carry out “deeper” perceptual processing would also be associated with the observed absence of later memory for features of the obstacle (Craik and Lockhart, 1972). The alert reader may have noticed that the Craik and Lockhart (1972) levels of processing model of memory was heavily influenced by Treisman’s (1964) view of attention!

I believe that Treisman’s general approach to how the attentional system is organized can also provide an explanation for aspects of the results reported by Scheiter et al. (2014). Their studies investigated the extent to which the provision of interesting but irrelevant information would distract individuals who were working to solve easy or difficult problems. Experiment 1 in the series showed no effects of distraction on performance given that the distracting information was entirely irrelevant to participants’ task and goals. However, Experiment 2 did show an effect of distraction when participants solved easy tasks; in this experiment participants were given a pending goal for future tasks, and the distracting information was relevant to this future goal. In Treisman’s terms, the pending goal would have the effect of setting favorable criteria for information relevant to the goal, thereby allowing the distracting information to be processed more fully and so consuming some portion of the individual’s limited attentional resources. This effect of a pending goal (see also Goschke and Kuhl, 1993) would thus lie somewhere between the very transient effects associated with sentence contexts (e.g., “the boy leaned out of the ____”) and the relatively permanent priming effects associated with stimuli such as the person’s own name. To summarize this point, maintaining a pending goal may be attention consuming in its own right, but may also function by enhancing the relevance of distracting information; both factors have the potential to reduce the level of current task performance.

One other interesting result reported by Scheiter et al. (2014) was that even with a pending goal, participants performing difficult tasks were able to resist distraction whereas those performing easy tasks were not. Results on this point are mixed, however. Earlier studies by Britton et al. (1983) found that *easier* text passages occupied more cognitive capacity than difficult passages in participants who also had to carry out a sensory RT task while reading. The common theme behind the two sets of results may be the degree to which the primary task “absorbs” attention and allows the participant to lock on to the task and thereby successfully combat distraction. Greater degrees of absorption may be associated with a variety of other variables such as interest and meaningfulness.

Beaman et al. (2014) explored the effects of auditory distraction on the recognition of word pairs. Interestingly, they looked at the effects of distraction on both recognition memory and also on the quality of responses as judged by confidence ratings, the proportions of answers withheld, and the proportions of correct judgments when answers were given. The main results were that distraction had a negative effect on both the straight cognitive aspect of recognition and the metacognitive aspects of how participants managed their decision-making. In this study the distracting materials were also words, so it seems possible that participants’ performance suffered both from having to block out the irrelevant distraction (a domain-general effect) and also from domain-specific effects associated with the confusion between target and distracting words. It was also the case that distraction occurred at both encoding and retrieval and this reduces somewhat the ability to analyze the locus of effects, as the authors acknowledge.

## OVERVIEW AND SUMMARY

The preceding articles cover a number of aspects of the problems (and occasional benefits) associated with the effects of distraction on cognitive performance. Some articles considered the benefits of EC conditions as a way to avoid the disruptive effects of distraction, and the consensus is that closing the eyes *is* beneficial under certain conditions. Both Mastroberardino and Vredeveldt (2014) and Kyriakidou et al. (2014) found that EC conditions increased the recall of visual details, although not of auditory details; Wais and Gazzaley (2014) showed that EC was beneficial for the recognition of visual objects, and Buchanan et al. (2014) showed that EC protected against the disruptive effects of social interactions. Rae and Perfect (2014) did not find an effect of dynamic visual noise on retrieval, but perhaps because the information to be retrieved (unrelated words) was qualitatively very different from the distracting material. One clear result regarding individual differences is that older adults are more vulnerable to the effects of distraction than are their younger counterparts (Hasher and Zacks, 1988) and this result was reported by Wais and Gazzaley (2014) and by Weeks and Hasher (2014). The latter article also illustrated the interesting corollary that older adults can actually derive benefits from the poorly inhibited distracting material – under certain conditions at least. The Wais and Gazzaley (2014) article emphasized the point that distraction can result in reduced fidelity of details retrieved from LTM. The idea that reduced attentional resources are associated with a reduction in recognition memory performance, and also in the accuracy of metacognitive monitoring of retrieval, was nicely illustrated in the study by Beaman et al. (2014). The article by Hyman et al. (2014) provided dramatic illustrations of how people can avoid obstacles yet remember few details of the objects later. I pointed out how these findings can be described in terms of Treisman’s (1964) “levels of analysis” view of selective attention, and suggested that Treisman’s views can also be used to understand the results of Scheiter et al. (2014). The basic point here is that some stimuli may inadvertently attract attention, thereby consuming some of the limited-capacity pool, and so interfere with the top-down management of ongoing task performance. Such cases of inadvertent attraction are most likely to occur when the perceptual system is tuned to expect the distracting stimulus – either due to the current context, highly meaningful stimuli maintained over the long term, or [as in the case described by Scheiter et al. (2014)] when stimuli are relevant to a goal being maintained for some future task. If the distracting stimulation requires responses (i.e., dual-task performance), more attention will be required and more disruption will ensue. Overall then, the effects of distraction likely depend on complex interactions among such factors as the attentional demands of the distracting information, the nature of the primary task, and the similarity of operations between those required by the primary task and those required to deal with the distracting information.

## Conflict of Interest Statement

The author declares that the research was conducted in the absence of any commercial or financial relationships that could be construed as a potential conflict of interest.
